# Supplementary material for: Independent Component Analysis Reveals the Transcriptional Regulatory Modules in Bradyrhizobium diazoefficiens USDA110
Source: Int J Mol Sci. 2023 Aug 8;24(16):12544. doi: 10.3390/ijms241612544 (PMC10454721; doi:10.3390/ijms241612544)
Supplement: Supplementary file 1 [file ijms-24-12544-s001.zip › Supplementary figures.pdf]

# Supplementary Figures

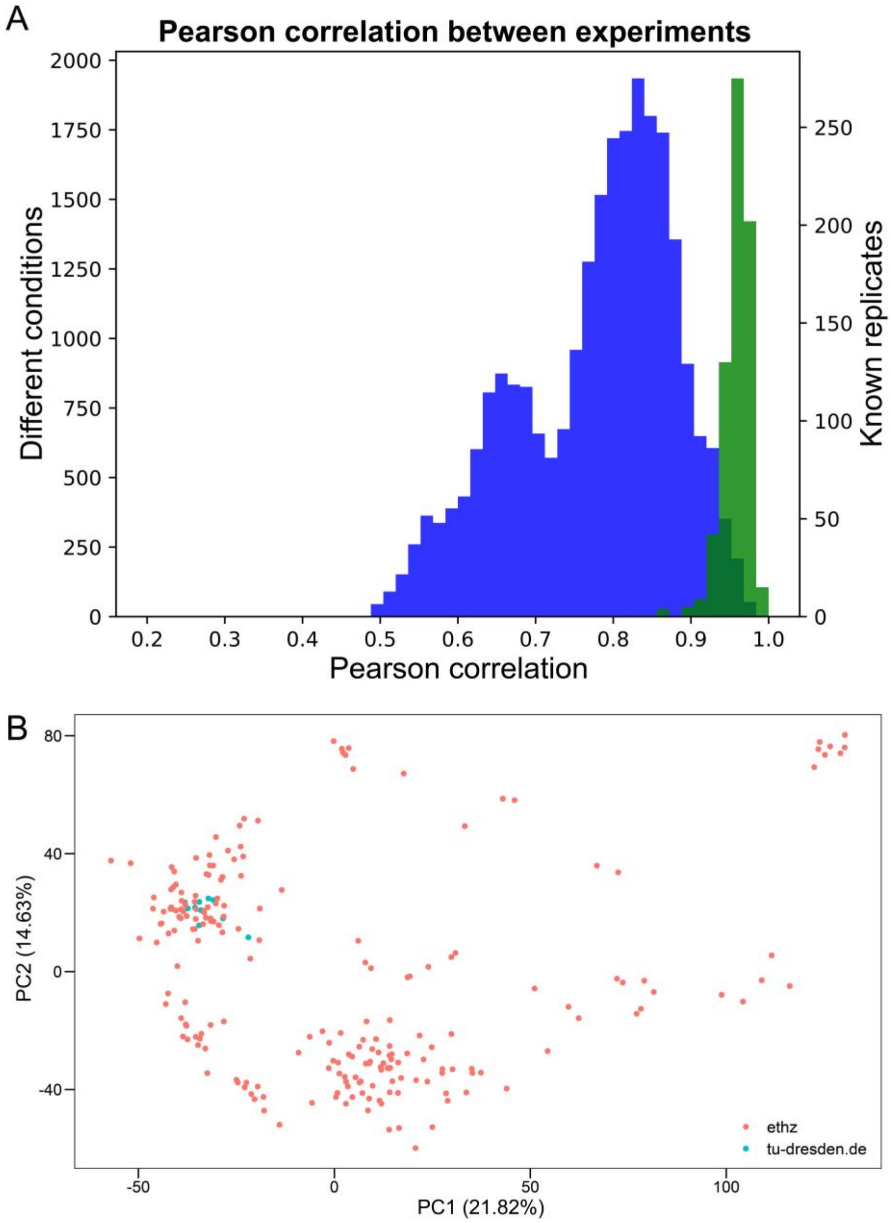


**Figure S1. Summary of data.** (A) Histogram of the Pearson correlation coefficient (PCC) between gene expression data, after centering to reference condition. (B) Loadings of the first two principal components (PC) of the compendium, colored by the researchers' institution. Red represents ethz (ETH Zurich) and blue represents tu-dresden (Dresden University of Technology). There were 216 samples from ethz or with ethz involved, and 10 samples from tu-dresden. The high coincidence of red and blue indicates high self-consistency.

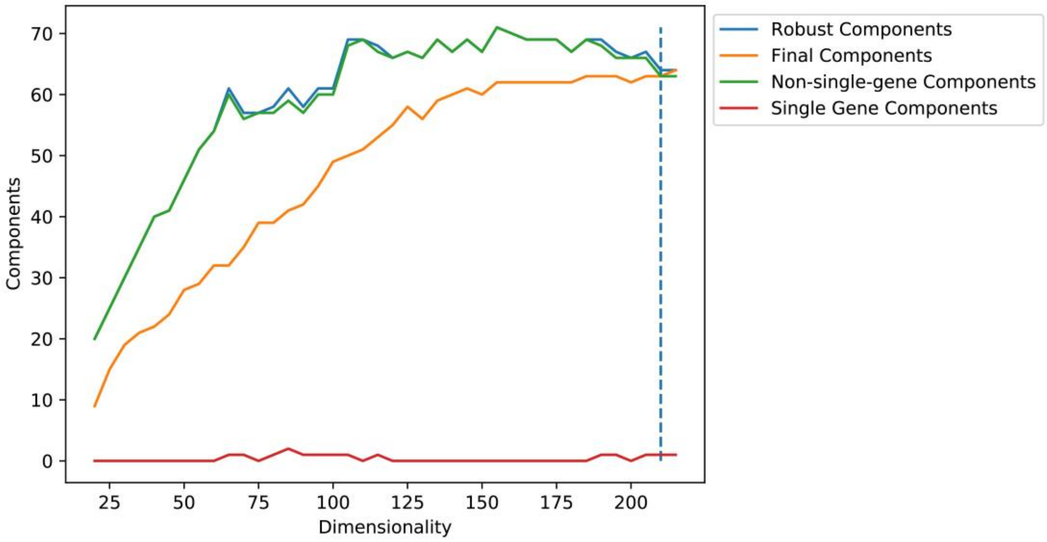


**Figure S2. Dimension analysis.** ICA was used in gene expression datasets and some independent components were obtained. As mentioned in **Materials and methods**, we performed clustering of the gene expression profile multiple times for dimensions between 20 and 215 with a step size of 5. All components computed from a multi-start ICA decomposition were counted as "robust components". A component was classified as "single gene" if the highest gene weight was more than twice the next highest; the number of non-single gene components was determined by subtracting the number of single gene components from the number of robust components [47].

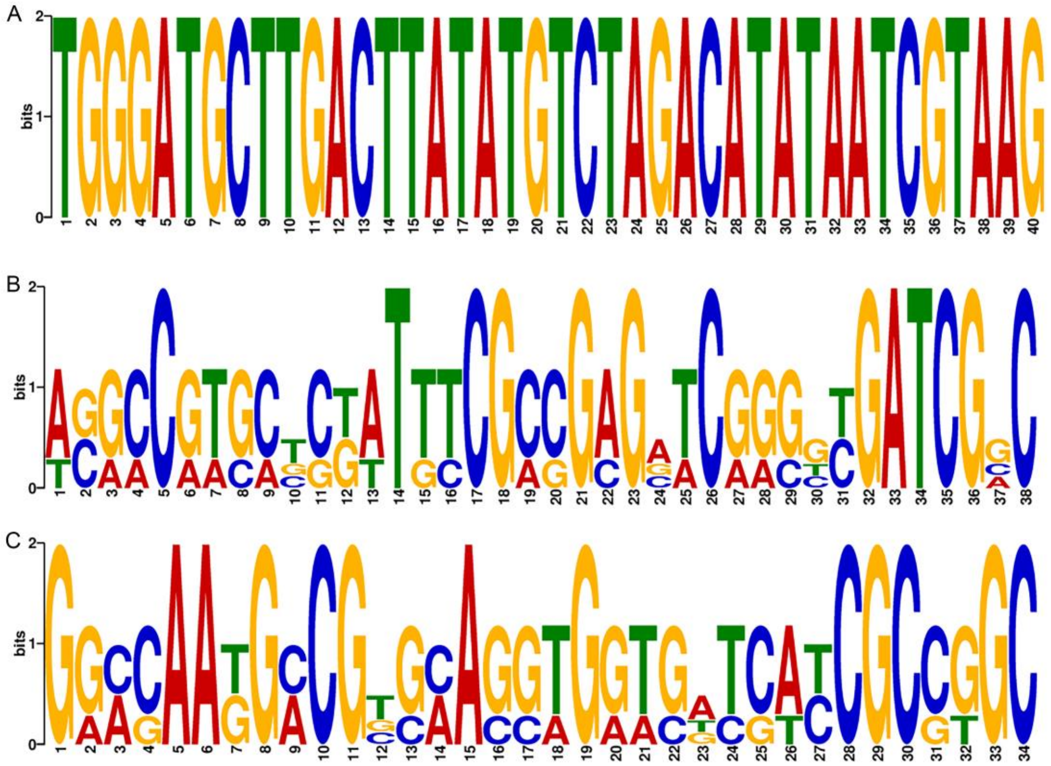


**Figure S3. The motifs identified in the upstream promoter regions of HutC iModulon genes.** (A) Motif-1. (B) Motif-2. (C) Motif-3.

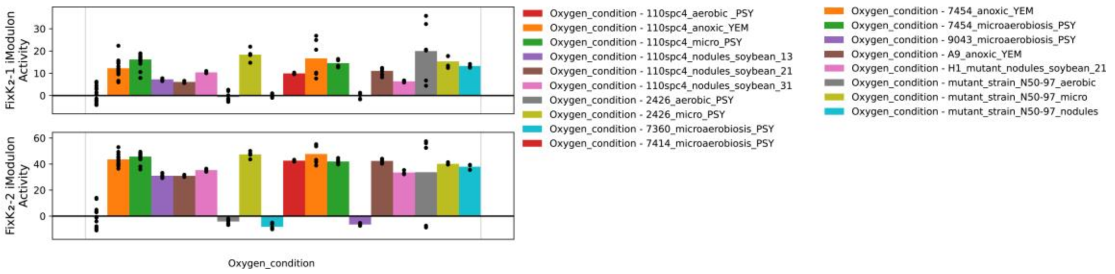


**Figure S4. The activities of FixK$_2$-1 iModulon and FixK$_2$-2 iModulon in response to oxygen conditions.** "110spc4_aerobic_PSY" represents wild-type strain cultured in PSY medium under aerobic conditions; "110spc4_anoxic_YEM" represents its cultivation in YEM medium under anoxic conditions; "110spc4_micro_PSY" represents the cultivation environment in PSY medium under microaerobic conditions; "110spc4_nodules_soybean_13", "110spc4_nodules_soybean_21", and "110spc4_nodules_soybean_31" represent 13 days, 21 days, and 31 days after the establishment of symbiosis between wild-type strain and legumes, respectively; "2426_aerobic_PSY" and "2426_micro_PSY" represent *regR* mutant strain cultured in PSY medium under aerobic conditions and microaerobic conditions, respectively; "7360_microaerobiosis_PSY" represents *fixj* mutant strain cultured in PSY medium under microaerobic conditions; "7414_microaerobiosis_PSY" represents *fixL* mutant strain cultured in PSY medium under microaerobic conditions; "7454_anoxic_YEM" represents *fixK$_1$* mutant strain cultured in YEM medium under anoxic conditions; "7454_microaerobiosis_PSY" represents *fixK$_1$* mutant strain cultured in PSY medium under microaerobic conditions; "9043_microaerobiosis_PSY" represents *fixK$_2$* mutant strain cultured in PSY medium under microaerobic conditions; "A9_anoxic_YEM" represents *nifA* mutant strain cultured in YEM medium under anoxic conditions; "H1_mutant_nodules_soybean_21" represents the establishment of symbiosis between *nifH* mutant strain and legumes; "mutant_strain_N50-97_aerobic" and "mutant_strain_N50-97_micro" represent *rpoN* mutant strain cultured in PSY medium under aerobic conditions and microaerobic conditions, respectively; "mutant_strain_N50-97_nodules" represents the condition of established symbiosis between *rpoN* mutant strain and legumes.

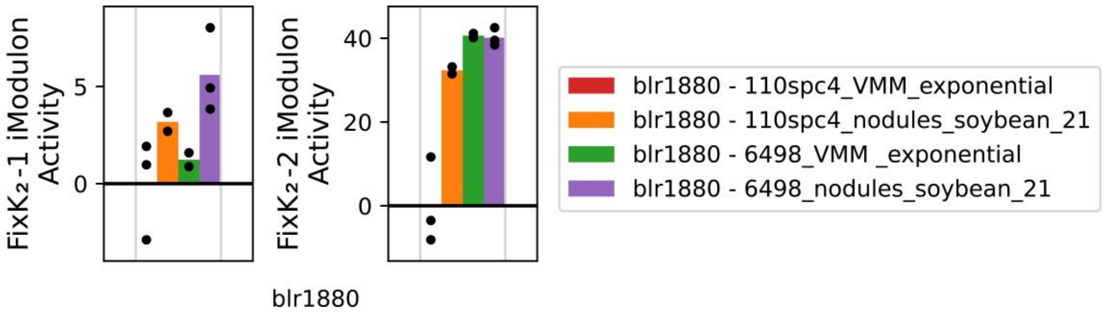


**Figure S5. FixK$_2$-1 iModulon and FixK$_2$-2 iModulon activities in the *blr1880* project.** "110spc4_VMM_exponential" represents the wild-type strain growing in VMM minimal medium; "110spc4_nodules_soybean_21" represents the wild-type strain growing with soybean; "6498_VMM_exponential" represents the *blr1880* mutant growing in VMM minimal medium; "6498_nodules_soybean_21" represents the *blr1880* mutant growing with soybean.
