# Supplementary material for: Independent Component Analysis Reveals the Transcriptional Regulatory Modules in Bradyrhizobium diazoefficiens USDA110
Source: Int J Mol Sci. 2023 Aug 8;24(16):12544. doi: 10.3390/ijms241612544 (PMC10454721; doi:10.3390/ijms241612544)
Supplement: Supplementary file 1 [file ijms-24-12544-s001.zip › Supplementary notes.pdf]

Supplementary Notes

The identified 64 iModulons

1. $FixK_2$-2 iModulon (n = 174, explained variance: 11.6%)

Category: Regulatory iModulon (MR and RR of 0.31 and 0.54, poorly-matched)

Inferred role: Oxidative adaptation

Related regulators: $FixK_2$

Top five genes with highest weights:

Locus tag	Gene weight	Gene name	Gene product
bsr5273	0.164988	bsr5273	hypothetical protein
bsr6066	0.140447	bsr6066	hypothetical protein
blr4638	0.132135	blr4638	unknown
blr4637	0.13115	blr4637	Hsp20/alpha crystallin family protein
bsr7036	0.12836	napE	periplasmic nitrate reductase%2C NapE protein

2. NifA/RpoN iModulon (n = 158, explained variance: 9.8%)

Category: Regulatory iModulon (MR and RR of 0.18 and 0.94, Regulon discovery)

Inferred role: Nitrogen fixation

Related regulators: NifA/RpoN

Top five genes with highest weights:

Locus tag	Gene weight	Gene name	Gene product
blr2106	0.173881	ectC	ectoine synthase
blr2143	0.162643	blr2143	cytochrome P450
blr2146	0.135722	blr2146	SDR family oxidoreductase
blr2132	0.128059	blr2132	MFS transporter
bsr1758	0.124131	bsr1758	unknown

3. **Null-2 iModulon (n = 0, explained variance: 9.1%)**
4. **Flagella iModulon (n = 46, explained variance: 4.1%)**

Category: Functional iModulon

Inferred role: Flagella synthesis

Related regulators: Unknown

Top five genes with highest weights:

Locus tag	Gene weight	Gene name	Gene product
bll6855	0.179196	flaF	flagellar biosynthesis regulator FlaF
bll6871	0.169093	flgI	flagellar basal body P-ring protein FlgI

bll6875	0.167821	flgC	flagellar basal body rod protein FlgC
bll6857	0.163486	flgK	flagellar hook-associated protein FlgK
bll6856	0.155349	flgL	flagellar hook-associated family protein

5. NnrR iModulon (n = 48, explained variance: 3.8%)

Category: Regulatory iModulon (MR and RR of 0.33 and 0.7, Regulon discovery)

Inferred role: Nitrogen metabolism

Related regulators: NnrR

Top five genes with highest weights:

Locus tag	Gene weight	Gene name	Gene product
blr3216	0.126556	norQ	CbbQ/NirQ/NorQ/GpvN family protein
blr3225	0.121794	blr3225	SDR family oxidoreductase
blr3212	0.120507	norE	cytochrome c oxidase subunit 3 family protein
blr3224	0.118598	blr3224	ABC transporter ATP-binding protein
blr3215	0.116001	norB	cbb3-type cytochrome c oxidase subunit I

6. T3SS iModulon (n = 45, explained variance: 3.6%)

Category: Functional iModulon

Inferred role: T3SS system

Related regulators: Unknown

Top five genes with highest weights:

Locus tag	Gene weight	Gene name	Gene product
bll1844	0.106808	bll1844	unknown
bll1804	0.106583	bll1804	hypothetical protein
bll2065	0.099323	icfA	carbonic anhydrase
bll2066	0.098801	bll2066	thermonuclease family protein
bll2047	0.097987	bll2047	unknown

7. Phenylalanine iModulon (n = 25, explained variance: 3.6%)

Category: Functional iModulon

Inferred role: Aromatic acid catabolism

Related regulators: Unknown

Top five genes with highest weights:

Locus tag	Gene weight	Gene name	Gene product
bll4766	0.13055	bll4766	TonB-dependent receptor

bll0332	0.119992	bll0332	transporter substrate-binding domain-containing protein
blr3167	0.114482	hyi	hydroxypyruvate isomerase
bll0333	0.10538	bll0333	PQQ-dependent methanol/ethanol family dehydrogenase
blr3278	0.098699	blr3278	unknown

8. Irr iModulon (n = 51, explained variance: 2.7%)

Category: Regulatory iModulon (MR and RR of 0.31 and 0.8, Regulon discovery)

Inferred role: Inorganic ion transport and metabolism

Related regulators: Irr

Top five genes with highest weights:

**Locus tag**	**Gene weight**	**Gene name**	**Gene product**
bsr3556	0.211146	bsr3556	unknown
bll4920	0.165991	bll4920	TonB-dependent siderophore receptor
bll7074	0.15866	hutX	heme utilization cystosolic carrier protein HutX
blr7296	0.157585	blr7296	hypothetical protein
blr3555	0.155132	fcuA	unknown

9. Null-3 iModulon (n = 0, explained variance: 2.2%)

10. Bacterial_chemotaxis iModulon (n = 163, explained variance: 2.1%)

Category: Functional iModulon

Inferred role: Multiple stress related response

Related regulators: Unknown

Top five genes with highest weights:

**Locus tag**	**Gene weight**	**Gene name**	**Gene product**
blr2344	0.078455	cheWII	chemotaxis protein CheW
bll4664	0.073031	bll4664	hypothetical protein
blr2539	0.068534	blr2539	cell wall hydrolase
bll6609	0.065634	bll6609	hypothetical protein
blr2194	0.06452	cheY	response regulator

11. XylR/bll3916 iModulon (n = 203, explained variance: 2.0%)

Category: Regulatory iModulon (MR and RR of 0.04 and 0.47, poorly-matched)

Inferred role: Sugar catabolism

Related regulators: XylR/bll3916

Top five genes with highest weights:

**Locus tag**	**Gene weight**	**Gene name**	**Gene product**

blr4886	0.112859	blr4886	ABC transporter permease
blr7868	0.091855	blr7868	sugar ABC transporter substrate-binding protein
blr7867	0.080423	blr7867	ABC transporter permease
bll4784	0.07756	bll4784	aldehyde dehydrogenase
blr6446	0.07659	blr6446	ABC transporter substrate-binding protein

12. Translation-2 iModulon (n = 4, explained variance: 1.8%)

Category: Functional iModulon

Inferred role: Protein synthesis

Related regulators: Unknown

Top four genes with highest weights:

**Locus tag**	**Gene weight**	**Gene name**	**Gene product**
blr4582	0.096446	argD	acetylornithine transaminase
bll5385	0.077776	rplF	50S ribosomal protein L6
bsl5891	0.077205	bsl5891	DUF3311 domain-containing protein
blr3719	-0.088141	blr3719	unknown

13. FixK$_2$-1 iModulon (n = 122, explained variance: 1.5%)

Category: Regulatory iModulon (MR and RR of 0.19 and 0.23, poorly-matched)

Inferred role: Oxidative adaptation

Related regulators: FixK$_2$

Top five genes with highest weights:

Locus tag	Gene weight	Gene name	Gene product
bll2515	0.118655	bll2515	PEP-utilizing enzyme
blr3464	0.100137	blr3464	phasin
blr7874	0.090421	blr7874	ABC transporter permease
blr3478	0.085555	blr3478	hypothetical protein
blr3482	0.078109	blr3482	hypothetical protein

14. Multiple_stress iModulon (n = 38, explained variance: 1.5%)

Category: Functional iModulon

Inferred role: Multiple stress response, Hydrogen peroxide

Related regulators: Unknown

Top five genes with highest weights:

Locus tag	Gene weight	Gene name	Gene product
bll3418	0.167848	bll3418	epoxide hydrolase

bll3417	0.162321	bll3417	carboxymuconolactone decarboxylase family protein
bll2772	0.155789	bll2772	cupin domain-containing protein
bll7429	0.14325	bll7429	sulfite exporter TauE/SafE family protein
bll3419	0.139741	bll3419	VOC family protein

15. Cell_mobility iModulon (n = 160, explained variance: 1.4%)

Category: Functional iModulon

Inferred role: Cell mobility

Related regulators: Unknown

Top five genes with highest weights:

Locus tag	Gene weight	Gene name	Gene product
blr7434	0.164645	blr7434	nuclear transport factor 2 family protein
blr5652	0.147191	blr5652	metalloregulator ArsR/SmtB family transcription factor
blr2836	0.125631	blr2836	ABC transporter permease
bll3089	0.120401	bll3089	MFS transporter
bsr4273	0.117379	bsr4273	hypothetical protein

16. Unchar-5 iModulon (n = 23, explained variance: 1.4%)

Category: Uncharacterized iModulon

Inferred role: Unknown

Related regulators: Unknown

Top five genes with highest weights:

Locus tag	Gene weight	Gene name	Gene product
bsl1637	0.197012	bsl1637	unknown
bll1858	0.172581	bll1858	effector protein NopP
bll2004	0.170716	bll2004	unknown
blr1880	0.160811	blr1880	LuxR family transcriptional regulator
blr1638	0.146932	blr1638	unknown

17. HrcA iModulon (n = 126, explained variance: 1.2%)

Category: Regulatory iModulon (MR and RR of 0.04 and 0.71, Regulon discovery)

Inferred role: Chaeperone

Related regulators: HrcA

Top five genes with highest weights:

Locus tag	Gene weight	Gene name	Gene product
blr5228	0.142238	blr5228	hypothetical protein

blr5229	0.138171	blr5229	unknown
blr5222	0.135983	blr5222	ETC complex I subunit
blr5233	0.130928	hspB	Hsp20 family protein
blr5234	0.127476	hspC	Hsp20/alpha crystallin family protein

18. bll2166 iModulon (n = 49, explained variance: 1.2%)

Category: Regulatory iModulon (MR and RR of 0.06 and 0.5, poorly-matched)

Inferred role: Amino acid transport

Related regulators: bll2166

Top five genes with highest weights:

Locus tag	Gene weight	Gene name	Gene product
bll7930	0.090562	bll7930	ABC transporter ATP-binding protein
blr2167	0.073383	blr2167	glucose 1-dehydrogenase
blr7589	0.063854	blr7589	indolepyruvate ferredoxin oxidoreductase family protein
bll7929	0.063778	bll7929	ABC transporter ATP-binding protein
blr2170	0.063525	blr2170	TRAP transporter substrate-binding protein

19. PhyR/EcfG-1 iModulon (n = 116, explained variance: 1.1%)

Category: Regulatory iModulon (MR and RR of 0.31 and 1.0, Regulon discovery)

Inferred role: Multiple stress

Related regulators: PhyR

Top five genes with highest weights:

Locus tag	Gene weight	Gene name	Gene product
blr7797	0.199154	blr7797	sigma-70 family RNA polymerase sigma factor
bll1466	0.192353	bll1466	DUF883 family protein
bll7795	0.164341	phyR	response regulator
bsr2400	0.163693	bsr2400	hypothetical protein
bsl1473	0.160273	bsl1473	CsbD family protein

20. Genomic-1 iModulon (n = 1, explained variance: 1.0%)

Category: Genomic iModulon

Inferred role: FixK deletion

Related regulators: Unknown

Weight of the only gene:

Locus tag	Gene weight	Gene name	Gene product
bll6061	0.113421	fixK	helix-turn-helix domain-containing protein

21. Unchar-19 iModulon (n = 13, explained variance: 1.0%)

Category: Uncharacterized iModulon

Inferred role: Unknown

Related regulators: Unknown

Top five genes with highest weights:

**Locus tag**	**Gene weight**	**Gene name**	**Gene product**
blr3719	0.116722	blr3719	unknown
bll0197	-0.064567	bll0197	branched-chain amino acid ABC transporter permease
bll7322	-0.064596	bll7322	Bax inhibitor-1/YccA family protein
blr7167	-0.065171	blr7167	hypothetical protein
bll0198	-0.071288	bll0198	amidase

22. Unchar-15 iModulon (n = 5, explained variance: 0.9%)

Category: Uncharacterized iModulon

Inferred role: Unknown

Related regulators: Unknown

Top five genes with highest weights:

Locus tag	Gene weight	Gene name	Gene product
bsl2713	0.067252	bsl2713	unknown
blr4630	0.060321	blr4630	hypothetical protein
bsl7703	0.058516	bsl7703	unknown
bll7668	0.058477	bll7668	unknown
blr5171	0.056941	blr5171	hypothetical protein

23. Translation-1 iModulon (n = 53, explained variance: 0.9%)

Category: Functional iModulon

Inferred role: Protein synthesis

Related regulators: Unknown

Top five genes with highest weights:

Locus tag	Gene weight	Gene name	Gene product
bsr6735	0.073068	pqqA	pyrroloquinoline quinone precursor peptide PqqA
bll5076	0.072999	bll5076	porin
bll5511	0.069656	bll5511	unknown
bll5085	0.068948	bll5085	unknown
blr4701	0.066887	blr4701	outer membrane beta-barrel protein

24. Copper iModulon (n = 24, explained variance: 0.8%)

Category: Functional iModulon

Inferred role: Metal related response

Related regulators: Unknown

Top five genes with highest weights:

Locus tag	Gene weight	Gene name	Gene product
bll4881	0.23423	bll4881	TonB-dependent receptor
bll4879	0.155451	bll4879	YcnI family protein
bll4880	0.142624	bll4880	copper chaperone PCu(A)C
bll4882	0.121918	bll4882	DUF2946 domain-containing protein
bll4878	0.120421	bll4878	CopD family protein

25. Sulfur-1 iModulon (n = 39, explained variance: 0.7%)

Category: Functional iModulon

Inferred role: Sulfur metabolism

Related regulators: Unknown

Top five genes with highest weights:

Locus tag	Gene weight	Gene name	Gene product
bll6452	0.182832	acd	acyl-CoA/acyl-ACP dehydrogenase
bll6455	0.18223	bll6455	ABC transporter substrate-binding protein
bll6454	0.158087	bll6454	ABC transporter permease subunit
bll7009	0.151427	ssuC	aliphatic sulfonate ABC transporter permease SsuC
bll7008	0.142211	bll7008	ATP-binding cassette domain-containing protein

26. Glyoxylate iModulon (n = 70, explained variance: 0.7%)

Category: Functional iModulon

Inferred role: Glyoxylate and dicarboxylate metabolism

Related regulators: Unknown

Top five genes with highest weights:

Locus tag	Gene weight	Gene name	Gene product
bll0904	0.255885	regR	ActR/PrrA/RegA family redox response regulator transcription factor
blr1516	0.177526	acrB	multidrug efflux RND transporter permease subunit
blr1515	0.170574	acrA	efflux RND transporter periplasmic adaptor subunit
bll6513	0.151516	bll6513	tripartite tricarboxylate transporter substrate binding protein BugD
bll2087	0.150236	bll2087	DUF5695 domain-containing protein

27. Denitrification iModulon (n = 11, explained variance: 0.6%)

Category: Functional iModulon

Inferred role: Denitrification

Related regulators: Unknown

Top five genes with highest weights:

Locus tag	Gene weight	Gene name	Gene product
bll2757	0.191972	$fixK_2$	helix-turn-helix domain-containing protein
bll4640	0.177432	bll4640	CBS domain-containing protein
blr1508	0.124139	blr1508	ABC transporter ATP-binding protein/permease
blr4641	0.122275	blr4641	c-type cytochrome
blr2231	0.104845	blr2231	unknown

28. Noise iModulon (n = 2, explained variance: 0.6%)

Category: Noise

29. T6SS iModulon (n = 43, explained variance: 0.5%)

Category: Functional iModulon

Inferred role: T6SS system

Related regulators: Unknown

Top five genes with highest weights:

Locus tag	Gene weight	Gene name	Gene product
bll1028	0.210422	carQ	sigma-70 family RNA polymerase sigma factor
bll2645	0.131034	bll2645	class I SAM-dependent methyltransferase
bsl0348	0.124052	bsl0348	hypothetical protein
blr7943	0.121305	blr7943	winged helix-turn-helix domain-containing protein
blr3596	0.115136	vgrG	type VI secretion system tip protein VgrG

30. Signal_transduction iModulon (n = 23, explained variance: 0.5%)

Category: Functional iModulon

Inferred role: Signal transduction mechanisms

Related regulators: Unknown

Top five genes with highest weights:

Locus tag	Gene weight	Gene name	Gene product
blr3174	0.078549	blr3174	flagellar basal body protein

bll7580	0.074057	bll7580	unknown
bsr5798	0.073848	bsr5798	unknown
blr0842	0.071932	blr0842	HD domain-containing protein
blr5569	0.071145	blr5569	transglutaminase-like cysteine peptidase

31. Sulfur-2 iModulon (n = 4, explained variance: 0.4%)

Category: Functional iModulon

Inferred role: Sulfur metabolism

Related regulators: Unknown

Top four genes with highest weights:

Locus tag	Gene weight	Gene name	Gene product
blr7556	0.091301	blr7556	alpha/beta hydrolase
bll5738	0.089557	bll5738	OsmC family protein
bll5736	0.08305	bll5736	sulfurtransferase
bll4570	0.072865	bll4570	sulfite reductase subunit alpha

32. Unchar-20 iModulon (n = 17, explained variance: 0.4%)

Category: Uncharacterized iModulon

Inferred role: Unknown

Related regulators: Unknown

Top five genes with highest weights:

Locus tag	Gene weight	Gene name	Gene product
bsr2110	0.081997	bsr2110	unknown
bsr3155	0.080973	bsr3155	hypothetical protein
bsr4694	0.072384	bsr4694	hypothetical protein
bll0046	0.070664	bll0046	DUF736 domain-containing protein
blr3553	0.066501	blr3553	hypothetical protein

33. Unchar-14 iModulon (n = 83, explained variance: 0.4%)

Category: Uncharacterized iModulon

Inferred role: Unknown

Related regulators: Unknown

Top five genes with highest weights:

Locus tag	Gene weight	Gene name	Gene product
bll6389	0.080399	bll6389	amino acid ABC transporter permease

bsl8075	0.077547	bsl8075	hypothetical protein
bsr4097	0.073936	bsr4097	hypothetical protein
blr8066	0.073484	blr8066	hypothetical protein
bsr7720	0.069323	bsr7720	unknown

34. Genomic-2 iModulon (n = 8, explained variance: 0.4%)

Category: Genomic iModulon

Inferred role: mutated nodW gene and an overexpressed nwsB gene

Related regulators: Unknown

Top five genes with highest weights:

**Locus tag**	**Gene weight**	**Gene name**	**Gene product**
bsr4258	0.182354	bsr4258	XapX domain-containing protein
blr4259	0.15384	blr4259	amidohydrolase
blr4260	0.151778	blr4260	DoxX family protein
blr4257	0.143049	blr4257	hydrolase
blr4261	0.128954	blr4261	hypothetical protein

35. NodD$_2$ iModulon (n = 29, explained variance: 0.4%)

Category: Regulatory iModulon (MR and RR of 0.24 and 1.0, poorly-matched)

Inferred role: Nodulation

Related regulators: $NodD_2$

Four genes with their weights:

Locus tag	Gene weight	Gene name	Gene product
blr2026	0.226006	nodB	chitooligosaccharide deacetylase NodB
blr2024	0.210995	nodY	unknown
blr2062	0.200278	noeI	FkbM family methyltransferase
bsl2015	0.171269	nolZ	unknown
blr2025	0.168993	nodA	NodA family N-acyltransferase

36. Unchar-12 iModulon (n = 29, explained variance: 0.3%)

Category: Uncharacterized iModulon

Inferred role: Unknown

Related regulators: Unknown

Top five genes with highest weights:

Locus tag	Gene weight	Gene name	Gene product

bll2704	0.162814	bll2704	hypothetical protein
bll6098	0.149958	bll6098	GNAT family N-acetyltransferase
bsl6101	0.14277	bsl6101	unknown
bll6102	0.133354	bll6102	unknown
bsl6099	0.113387	bsl6099	hypothetical protein

37. Leucine iModulon (n = 30, explained variance: 0.3%)

Category: Functional iModulon

Inferred role: Leucine degradation

Related regulators: Unknown

Top five genes with highest weights:

Locus tag	Gene weight	Gene name	Gene product
bll0889	0.174453	bll0889	MHS family MFS transporter
bll0888	0.106729	bll0888	acyl CoA:acetate/3-ketoacid CoA transferase
bll0883	0.086151	bll0883	branched-chain amino acid ABC transporter permease
bll0884	0.077493	bll0884	branched-chain amino acid ABC transporter permease
bll8294	0.071676	bll8294	hypothetical protein

38. BioR iModulon (n = 31, explained variance: 0.3%)

Category: Regulatory iModulon (MR and RR of 0.16 and 0.83, Regulon discovery)

Inferred role: Biotin metabolism

Related regulators: BioR

Top five genes with highest weights:

Locus tag	Gene weight	Gene name	Gene product
bll3379	0.15108	bll3379	SRPBCC family protein
bll3384	0.124098	bll3384	branched-chain amino acid ABC transporter permease
bll3376	0.123794	bll3376	unknown
bll3383	0.117503	bll3383	ABC transporter ATP-binding protein
bll3374	0.108801	bll3374	molybdopterin-dependent oxidoreductase

39. bll3348 iModulon (n = 15, explained variance: 0.3%)

Category: Functional iModulon

Inferred role: Amino acid transportation

Related regulators: bll3348

Top five genes with highest weights:

Locus tag	Gene weight	Gene name	Gene product
blr3351	0.256889	cutM	FAD binding domain-containing protein
blr3354	0.235104	blr3354	branched-chain amino acid ABC transporter permease
blr3357	0.195327	blr3357	ABC transporter ATP-binding protein
blr3355	0.179847	blr3355	branched-chain amino acid ABC transporter permease
blr3353	0.171464	blr3353	ABC transporter substrate-binding protein

40. Unchar-23 iModulon (n = 10, explained variance: 0.3%)

Category: Uncharacterized iModulon

Inferred role: Unknown

Related regulators: Unknown

Top five genes with highest weights:

Locus tag	Gene weight	Gene name	Gene product
bll1028	0.130941	carQ	sigma-70 family RNA polymerase sigma factor
bsr4694	0.114292	bsr4694	hypothetical protein
bll6615	0.099421	bll6615	DUF983 domain-containing protein
blr2475	0.097122	tspO	tryptophan-rich sensory protein
blr2474	0.091157	blr2474	fasciclin domain-containing protein

41. Unchar-3 iModulon (n = 62, explained variance: 0.3%)

Category: Uncharacterized iModulon

Inferred role: Unknown

Related regulators: Unknown

Top five genes with highest weights:

**Locus tag**	**Gene weight**	**Gene name**	**Gene product**
bll1605	0.12884	bll1605	unknown
bll1762	0.116638	bll1762	unknown
bsl7758	0.103917	bsl7758	unknown
bsl3175	0.102297	bsl3175	unknown
bsr6130	0.096649	bsr6130	unknown

42. Calvin_cycle iModulon (n = 9, explained variance: 0.3%)

Category: Functional iModulon

Inferred role: Calvin_cycle

Related regulators: Unknown

Top five genes with highest weights:

**Locus tag**	**Gene weight**	**Gene name**	**Gene product**

blr2588	0.190628	rpe	ribulose-phosphate 3-epimerase
blr2581	0.180744	cbbF	class 1 fructose-bisphosphatase
blr2585	0.161435	cbbL	form I ribulose bisphosphate carboxylase large subunit
blr2586	0.158164	cbbS	ribulose bisphosphate carboxylase small subunit
blr2583	0.157865	tkt	transketolase

43. HutC iModulon (n = 10, explained variance: 0.3%)

Category: Regulatory iModulon (MR and RR of 0.8 and 1.0, well-matched)

Inferred role: Histidine metabolism

Related regulators: HutC

Top five genes with highest weights:

Locus tag	Gene weight	Gene name	Gene product
bll6243	0.232622	hutI	imidazolonepropionase
bll6241	0.210829	hutU	urocanate hydratase
blr6246	0.20028	blr6246	ABC transporter substrate-binding protein
blr6244	0.193763	blr6244	formimidoylglutamate deiminase
blr6247	0.192148	blr6247	ABC transporter permease

44. Oxidation_stress iModulon (n = 21, explained variance: 0.2%)

Category: Functional iModulon

Inferred role: Oxidative stress

Related regulators: Unknown

Top five genes with highest weights:

Locus tag	Gene weight	Gene name	Gene product
bll1026	0.250415	bll1026	thioredoxin family protein
bll1027	0.249054	bll1027	cytochrome c biogenesis CcdA family protein
blr3038	0.246784	SigD	sigma-70 family RNA polymerase sigma factor
blr7741	0.232377	blr7741	alpha/beta hydrolase
bll6527	0.193172	bll6527	DUF417 family protein

45. Unchar-17 iModulon (n = 16, explained variance: 0.2%)

Category: Uncharacterized iModulon

Inferred role: Unknown

Related regulators: Unknown

Top five genes with highest weights:

Locus tag	Gene weight	Gene name	Gene product
blr1812	0.072681	nolB	hypothetical protein
blr6531	0.071433	blr6531	cyclophilin-like fold protein
blr0947	0.068686	mtgA	monofunctional biosynthetic peptidoglycan transglycosylase
blr3479	0.068274	blr3479	hypothetical protein
blr1658	0.067726	blr1658	unknown

46. Methane iModulon (n = 14, explained variance: 0.2%)

Category: Functional iModulon

Inferred role: Methane oxidation

Related regulators: Unknown

Top five genes with highest weights:

Locus tag	Gene weight	Gene name	Gene product
blr3678	0.180101	blr3678	FAD-binding oxidoreductase
blr3679	0.167302	blr3679	aromatic/alkene monooxygenase hydroxylase subunit beta
blr3681	0.166659	blr3681	amidohydrolase
blr3675	0.157368	blr3675	NAD(P)-dependent alcohol dehydrogenase
blr3677	0.144864	blr3677	aromatic/alkene/methane monooxygenase hydroxylase/oxygenase subunit alpha

47. Unchar-13 iModulon (n = 21, explained variance: 0.2%)

Category: Uncharacterized iModulon

Inferred role: Unknown

Related regulators: Unknown

Top five genes with highest weights:

**Locus tag**	**Gene weight**	**Gene name**	**Gene product**
blr4764	0.088969	blr4764	unknown
bsr0027	0.08018	bsr0027	unknown
blr6967	0.070714	blr6967	MFS transporter
bll0046	0.062611	bll0046	DUF736 domain-containing protein
bsl8265	0.062461	bsl8265	hypothetical protein

48. Unchar-11 iModulon (n = 36, explained variance: 0.2%)

Category: Uncharacterized iModulon

Inferred role: Unknown

Related regulators: Unknown

Top five genes with highest weights:

Locus tag	Gene weight	Gene name	Gene product
blr4638	0.119363	blr4638	unknown
blr4641	0.0989	blr4641	c-type cytochrome
bll4639	0.097273	bll4639	trypsin-like peptidase domain-containing protein
blr4652	0.094253	blr4652	nitroreductase
bll8248	0.089078	bll8248	unknown

49. Null-1 iModulon (n = 0, explained variance: 0.2%)

50. Unchar-21 iModulon (n = 18, explained variance: 0.2%)

Category: Uncharacterized iModulon

Inferred role: Unknown

Related regulators: Unknown

Top five genes with highest weights:

Locus tag	Gene weight	Gene name	Gene product
bll2172	0.067897	bll2172	SMP-30/gluconolactonase/LRE family protein
blr4764	0.064078	blr4764	unknown
blr8233	0.061553	blr8233	unknown
bll8177	0.060331	bll8177	AAA family ATPase

bll8212	0.058562	bll8212	FAD-dependent oxidoreductase

51. Genomic-3 iModulon (n = 13, explained variance: 0.1%)

Category: Genomic iModulon

Inferred role: Unknown

Related regulators: Unknown

Top five genes with highest weights:

Locus tag	Gene weight	Gene name	Gene product
bsl8208	0.558865	bsl8208	unknown
bll1684	0.08263	bll1684	hypothetical protein
bll5216	0.068854	bll5216	unknown
bsl5490	0.065947	bsl5490	hypothetical protein
blr3564	0.06178	blr3564	unknown

52. Unchar-16 iModulon (n = 35, explained variance: 0.1%)

Category: Uncharacterized iModulon

Inferred role: Unknown

Related regulators: Unknown

Top five genes with highest weights:

Locus tag	Gene weight	Gene name	Gene product
bsr3027	0.082104	bsr3027	unknown
bsr8005	0.080878	bsr8005	unknown
bll8308	0.075974	bll8308	hypothetical protein
blr3702	0.072975	blr3702	hypothetical protein
bll8227	0.072579	bll8227	unknown

53. Unchar-9 iModulon (n = 64, explained variance: 0.1%)

Category: Uncharacterized iModulon

Inferred role: Unknown

Related regulators: Unknown

Top five genes with highest weights:

Locus tag	Gene weight	Gene name	Gene product
bsr1982	0.098423	bsr1982	unknown
bll0069	0.075158	bll0069	ImmA/IrrE family metallo-endopeptidase
bll5145	0.074028	bll5145	unknown

bll6516	0.064643	bll6516	chromate transporter
blr3548	0.062723	blr3548	hypothetical protein

54. Unchar-1 iModulon (n = 58, explained variance: 0.1%)

Category: Uncharacterized iModulon

Inferred role: Unknown

Related regulators: Unknown

Top five genes with highest weights:

Locus tag	Gene weight	Gene name	Gene product
blr4764	0.087665	blr4764	unknown
bsl1111	0.087548	bsl1111	hypothetical protein
blr3553	0.08388	blr3553	hypothetical protein
bll7174	0.078052	bll7174	TetR/AcrR family transcriptional regulator
blr0854	0.077321	blr0854	GNAT family N-acetyltransferase

55. Unchar-10 iModulon (n = 28, explained variance: 0.1%)

Category: Uncharacterized iModulon

Inferred role: Unknown

Related regulators: Unknown

Top five genes with highest weights:

**Locus tag**	**Gene weight**	**Gene name**	**Gene product**
bll8287	0.08176	trbC	TrbC/VirB2 family protein
bll6552	0.073243	bll6552	DoxX family protein
bsl2999	0.073104	bsl2999	unknown
blr2827	0.062721	blr2827	TRAP transporter substrate-binding protein
bll0075	0.062298	bll0075	restriction endonuclease

56. Unchar-4 iModulon (n = 2, explained variance: 0.1%)

Category: Uncharacterized iModulon

Inferred role: Unknown

Related regulators: Unknown

Two genes with their weights:

**Locus tag**	**Gene weight**	**Gene name**	**Gene product**
bsr1621	0.086129	bsr1621	DUF2274 domain-containing protein
bll1960	0.083093	bll1960	ribbon-helix-helix protein%2C CopG family

57. PhyR/EcfG-2 iModulon (n = 14, explained variance: 0.1%)

Category: Regulatory iModulon (MR and RR of 0.36 and 0.14, poorly-matched)

Inferred role: Thermal and dessication stress related response

Related regulators: PhyR

Top five genes with highest weights:

**Locus tag**	**Gene weight**	**Gene name**	**Gene product**
bll7795	0.248598	phyR	response regulator
bll3764	0.067877	bll3764	unknown
blr0028	0.067839	blr0028	acetoacetate decarboxylase family protein
blr1196	0.063085	ohr	organic hydroperoxide resistance protein
bll8295	0.062501	bll8295	ABC transporter ATP-binding protein/permease

58. AA_transport iModulon (n = 24, explained variance: 0.1%)

Category: Functional iModulon

Inferred role: Branched amino acid transportation

Related regulators: Unknown

Top five genes with highest weights:

Locus tag	Gene weight	Gene name	Gene product
blr2924	0.167438	blr2924	branched-chain amino acid ABC transporter permease
blr2921	0.161754	blr2921	putative hydro-lyase
blr2926	0.157678	blr2926	ABC transporter ATP-binding protein
blr2923	0.147455	blr2923	branched-chain amino acid ABC transporter permease
blr2925	0.142878	blr2925	ABC transporter ATP-binding protein

59. Unchar-2 iModulon (n = 18, explained variance: 0.1%)

Category: Uncharacterized iModulon

Inferred role: Unknown

Related regulators: Unknown

Top five genes with highest weights:

Locus tag	Gene weight	Gene name	Gene product
bsr4178	0.094004	bsr4178	unknown
bsr1758	0.069289	bsr1758	unknown
blr4654	0.069178	blr4654	hypothetical protein
blr3565	0.068757	blr3565	unknown
bll8223	0.067656	bll8223	hypothetical protein

60. Unchar-7 iModulon (n = 124, explained variance: 0.1%)

Category: Uncharacterized iModulon

Inferred role: Unknown

Related regulators: Unknown

Top five genes with highest weights:

**Locus tag**	**Gene weight**	**Gene name**	**Gene product**
blr8204	0.098849	blr8204	unknown
blr3612	0.08525	blr3612	unknown
blr8022	0.076594	ligD	non-homologous end-joining DNA ligase
bll6915	0.072148	bll6915	Gfo/Idh/MocA family oxidoreductase
bll8212	0.065906	bll8212	FAD-dependent oxidoreductase

61. Unchar-22 iModulon (n = 18, explained variance: 0.1%)

Category: Uncharacterized iModulon

Inferred role: Unknown

Related regulators: Unknown

Four genes with their weights:

Locus tag	Gene weight	Gene name	Gene product
bsl3676	0.097353	bsl3676	unknown
bsr0036	0.082354	bsr0036	unknown
bll0025	0.078422	bll0025	oleate hydratase
blr8271	0.077065	blr8271	helix-turn-helix transcriptional regulator

62. Unchar-6 iModulon (n = 41, explained variance: 0.1%)

Category: Uncharacterized iModulon

Inferred role: Unknown

Related regulators: Unknown

Top five genes with highest weights:

Locus tag	Gene weight	Gene name	Gene product
bsl4812	0.084809	bsl4812	Flp family type IVb pilin
bll3228	0.071785	bll3228	LysR substrate-binding domain-containing protein
blr6578	0.070588	blr6578	ABC transporter permease
bll2637	0.069633	bll2637	DUF190 domain-containing protein
bsr2151	0.067647	bsr2151	unknown

63. Unchar-8 iModulon (n = 53, explained variance: 0.1%)

Category: Uncharacterized iModulon

Inferred role: Unknown

Related regulators: Unknown

Top five genes with highest weights:

**Locus tag**	**Gene weight**	**Gene name**	**Gene product**
blr4630	0.089313	blr4630	hypothetical protein
bll0415	0.076613	leuD	3-isopropylmalate dehydratase small subunit
bsl1708	0.075724	bsl1708	unknown
bll2661	0.075163	bll2661	DUF2721 domain-containing protein
blr1731	0.071016	hypB	unknown

64. Unchar-18 iModulon (n = 83, explained variance: 0.1%)

Category: Uncharacterized iModulon

Inferred role: Unknown

Related regulators: Unknown

Top five genes with highest weights:

**Locus tag**	**Gene weight**	**Gene name**	**Gene product**

bll1836	0.093132	bll1836	unknown
bll8055	0.092659	bll8055	hypothetical protein
blr3358	0.088928	blr3358	Rieske (2Fe-2S) protein
bsr0350	0.079721	bsr0350	unknown
bsl8315	0.079154	bsl8315	DUF3892 domain-containing protein
